# Changes in relative histone abundance and heterochromatin in αA-crystallin and αB-crystallin knock-in mutant mouse lenses

**DOI:** 10.1186/s13104-020-05154-7

**Published:** 2020-07-02

**Authors:** Usha P. Andley, Brittney N. Naumann, Paul D. Hamilton, Stephanie L. Bozeman

**Affiliations:** Department of Ophthalmology and Visual Sciences, Washington University School of Medicine, 660 South Euclid Avenue, Campus Box 8096, St. Louis, MO 63110 USA

**Keywords:** Crystallin, Cataract, Mouse lens, Histones, Mass spec, Electron microscopy

## Abstract

**Objective:**

Understanding the mechanisms of cataract formation is important for age-related and hereditary cataracts caused by mutations in lens protein genes. Lens proteins of the crystallin gene families α-, β-, and γ-crystallin are the most abundant proteins in the lens. Single point mutations in crystallin genes cause autosomal dominant cataracts in multigenerational families. Our previous proteomic and RNAseq studies identified genes and proteins altered in the early stages of cataract formation in mouse models. Histones H2A, H2B, and H4 increase in abundance in αA- and αB-crystallin mutant mouse lenses and in cultured cells expressing the mutant form of αA-crystallin linked with hereditary cataracts.

**Results:**

In this study of histones in mutant lenses, we extracted histones from adult mouse lenses from *cryaa*-R49C and *cryab*-R120G mutant knock-in mice. We characterized the histones using matrix-assisted laser desorption/ionization time of flight (MALDI-TOF)-mass spectrometric analysis and gel electrophoresis and characterized the lens nucleus morphology using electron microscopy (EM). The relative abundance of histone H3 protein decreased in lenses from *cryaa*-R49C mutant mice and the relative abundance of histone H2 increased in these lenses. Electron microscopy of nuclei from *cryaa*-R49C-homozygous mutant mouse lenses revealed a pronounced alteration in the distribution of heterochromatin.

## Introduction

The ocular lens consists of a single layer of lens epithelial cells on its anterior surface that overlay the lens fiber cells. The lens is encapsulated by a unique basement membrane (i.e., the capsule) that surrounds the entire lens [1]. The lens epithelial cells in the equatorial region undergo terminal differentiation into elongated fiber cells that contain very high concentrations of crystallin proteins. Alpha, β, and γ are the three families of crystallins in mammalian lenses [2, 3]. During aging, lens proteins undergo significant post-translational modifications that result in lens cataract formation. These cataracts are common in individuals > 70 years of age [4]. Mutations in lens protein genes, including the crystallin genes, are associated with autosomal dominant cataract formation [5, 6]. Human patients harboring certain point mutations in αA- and αB-crystallin develop cataracts as juveniles [7, 8]. One *cryaa* gene mutation (i.e., cryaa-R49C arginine to glycine) is present in four generations of a Caucasian family [7]. Individuals heterozygous (het) for the mutant allele have early onset cataract formation. The mechanisms of autosomal dominant cataract formation may be elucidated using knock-in mouse models in which the mutant gene replaces the normal gene in every cell [9, 10]. The *cryaa*-R49C homozygous (homo) mice have cataracts at birth that are often associated with a small eye and small lens phenotype. Analysis of protein and gene expression changes in these mutant lenses found an increased expression of histone proteins and genes, suggesting cataracts and histone gene expression are related [11, 12].

The chromatin core comprises four histone core proteins (H2A, H2B, H3, and H4). Chromatin consists of a repeating unit of nucleosomes. The DNA double helix makes two turns around the octamer of four histones (H3:H4)_2_(H2A:H2B)_2_. The histone amino terminal tails play a critical role in control of gene expression. Certain histone modifications are enriched in either actively transcribed or repressed chromatin regions [13]. The acetylation of H3 and H4 has an important role in gene regulation [14]. Histone acetylation lowers superoxide dismutase expression in age-related cataracts [14]. Inhibiting histone deacetylation inhibits oxidative stress in the human lens epithelial cell line HLE B-3 [15]. Histone methylation is also a key regulator of lens development via its binding with transcriptional factors, and histone methyltransferases associate with Pax 6, a key regulator of ocular lens development [16]. Mutations in *hist2h3c1* result in perturbed eye development and a small eye phenotype [17]. No studies have used mouse models to examine effects of cataract development on alterations in abundance of histones extracted from lenses. We designed this study to examine histone expression in extracts of mouse lenses. To better understand the relationship between histones and cataract in *cryaa*-R49C knock-in mutant mice, we extracted histones from wild-type (WT) and cataractous mouse lenses and analyzed them using MALDI mass spectroscopy and gel electrophoresis.

## Main text

### Methods

Adult het and homo knock-in mice carrying the αA-crystallin R49C mutation (*cryaa*-R49C) and the αB-crystallin R120G mutation (*cryab*-R120G) on a C57Bl/6 J background were generated for this study [9, 10]. Six to eight mice per genotype were used for histone extraction. Age-matched C57BL/6J WT mice were used as controls. Mice were euthanized using CO_2_ inhalation. The Institutional Animal Care and Use Committee at Washington University approved all experimental procedures using the mice.

Histones were extracted from adult mouse lenses using a modified version of previous protocols [18, 19]. Briefly, 12–16 lenses from each mouse genotype were extracted on ice in PBS containing a protease inhibitor cocktail and a hypotonic lysis buffer (10 mM Tris–HCL, pH 8.0; 1.5 mM MgCl_2_; 1 mM KCl; 0.1 mM DTT; 3 µM trichostatin A; phenylmethylsulfonyl fluoride). The lenses were vortexed, homogenized, and incubated for 30 min at 4 °C in hypotonic lysis buffer, followed by centrifugation at 10,000×*g* for 10 min. The resulting supernatant was vortexed after the addition of 0.4 N H_2_SO_4_, and then incubated overnight at 4 °C. After centrifugation at 10,000×*g* for 10 min, the histone-containing supernatant was treated with trifluoroacetic acid, incubated overnight on ice, and then centrifuged to precipitate the histones. The resulting histone pellet was washed with ice-cold acetone, air-dried, and suspended in 80 μl deionized water to obtain the histone preparation.

The histone extracts were dried on a SpeedVac concentrator, resuspended in 5 µl deionized water, and subjected to MALDI-TOF MS analysis. Histones extracted from mouse lenses were analyzed using reverse phase HPLC (RP-HPLC). Acid-extracted histones were run on a reverse-phase RP-300 Aquapore Octyl C8 column (22 cm long and 4.6 mm internal diameter) with an acetonitrile gradient. A 50-µl sample loop and an Agilent Technologies HPLC system 1220 Infinity LC equipped with a variable wavelength detector with pumps, UV detector, and fraction collector were used for the HPLC. Data were collected in a Bruker ultrafleXtreme instrument and analyzed using flexAnalysis software version 3.4. The MALDI-TOF data represented the sum of 8 or 9 laser shots obtained using the LP_5-50_kDa.par linear positive mode method. Bruker calibration standard proteins were also analyzed. The peaks were identified by reference to previously published studies [20–22].

Sodium dodecyl sulfate polyacrylamide gel electrophoresis (SDS-PAGE) on extracted histones was performed using 10–20% Tris–glycine gels (Life Technologies). Pre-stained molecular weight markers (Invitrogen) were used. Histones were at room temperature in electrophoresis buffer before loading on the gels. Proteins were analyzed by Coomassie blue staining and immunoblotting.

Eyes were enucleated and lenses were extracted from WT, *cryaa*-R49C-het, *cryaa*-R49C-homo, *cryab*-R120G-het, and *cryab*-R120G-homo mice. Electron microscopy was performed on lens sections to examine chromatin in nuclei of lens epithelial and fiber cells as described previously [23].

## Results

**Fig. 1:**
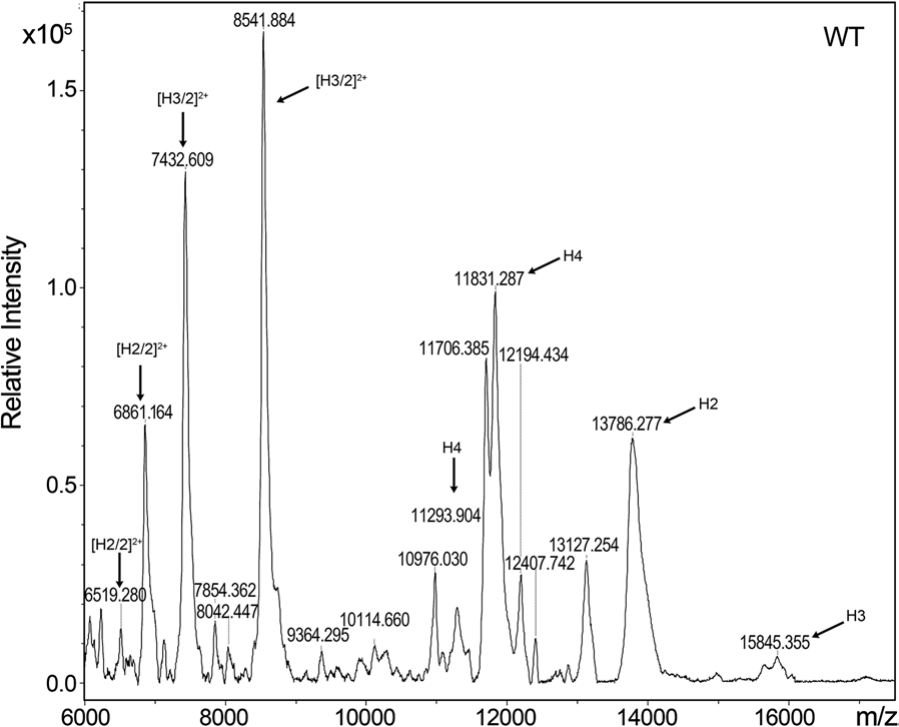
MALDI-TOF MS analysis of histones isolated from adult mouse lenses. WT, wild type. The histone peaks were identified based on their mass to charge (m/z) ratios. The m/z peaks on the left side of the x-axis represent doubly-charged ions for histones H4 [H4/2]^2+^, H2 [H2/2]^2+^, and H3 [H3/2]^2+^, respectively. MALDI spectra usually deliver single charged ions, but can also produce doubly-charged ions, especially for proteins

**Fig. 2:**
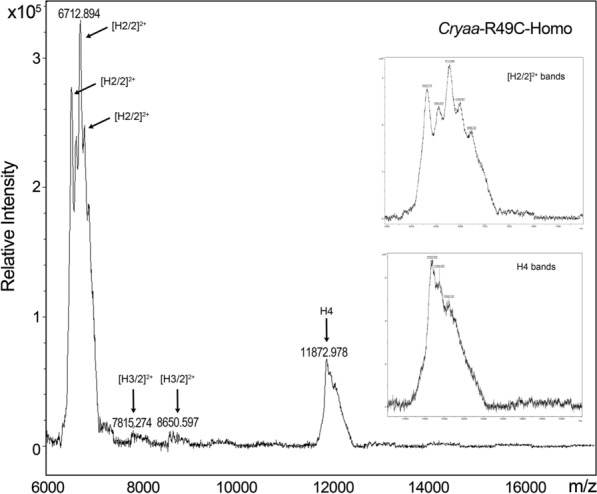
MALDI-TOF MS analysis of histones isolated from *cryaa*-R49C-homo mouse lenses (related to Fig. 1). The insets show the spectra of the H2, H3, and H4 histones in detail

Previously we reported the effects of these mutations on lens opacification and observed prominent opacities in the lens nucleus, and morphology changes in lens epithelial and fiber cells [10, 23, 24]. Previous work focused on proteomic and RNAseq analyses of 2-day-old mouse lenses with in vivo cataracts [11, 12]. Here, we extracted histones from adult mouse lenses from 3–4 months old mice and used MALDI-TOF to identify histones H2A, H2B, H3, and H4 in the extracts. Histone composition was qualitatively similar in the lenses from WT, *cryaa*-R49C-het, *cryaa*-R49C-homo, *cryab*-R120G-het, and *cryab*-R120G-homo mice and was similar to bovine histones [20]. However, analysis of the relative intensities of the histone peaks in the mass spectra revealed significant changes in the H2A/H2B and H3/H4 ratios (Additional files 1: Table S1 and 2: Table S2). These histone pairs form the nucleosome. Histone H3 was the most abundant histone in WT mouse lens histone extracts followed by H4 (Fig. 1). The H3/H4 ratio was 1.06 in histones extracted from WT lenses. In *cryaa*-R49C-het lenses, we extracted histones H3 and H4 as the most abundant histone, with an H3/H4 ratio of 1.009 (Additional file 2: Table S2 and Additional file 3: Figure S1). In *cryaa*-R49C-homo lens histone extracts, histone H2 was the most abundant followed by histone H4 (Fig. 2). Histone H3 was extremely low in these lenses. In the *cryab*-R120G-het lens histone extracts, histone H4 was the most abundant followed by histone H3 and H2 (Fig. 2 and Additional files 2: Table S2, 4:  Figure S2). In contrast, histone H3 was the most abundant in the *cryab*-R120G-homo lenses, followed by H4 and H2 (Additional file 5: Figure S3).

Quantitative analysis results of histone mass spectral data are presented in Additional file 2: Table S2. The decrease in the H3/H4 ratio found in *cryaa*-R49C-homo and *cryab*-R120G-het lenses was due to a decrease in the abundance of H3. In *cryaa*-R49C-homo lenses, the major change was the loss of [H3/2]^2+^ and an increase in the relative abundance of [H2]^2+^. In *cryab*-R120G homo lenses, the major change was the loss of H4 and an increase in [H3/2]^2+^. The relative proportion of H2A decreased substantially in *cryaa*-R49C-het, *cryab*-R120G-het and -homo lenses relative to WT lenses.

Histones extracted from lenses obtained from 3- to 4-month-old WT and *cryaa*-R49C-het mice were analyzed (Additional file 6: Figure S4). Compared with WT mice, the SDS-PAGE analysis found that in histones extracted from the lenses of *cryaa*-R49C-het mice, the antibody to histone H2B detected an increased amount of a protein band migrating with mobility at ~ 17 kDa. Previous proteomic studies suggest that histone fragments crosslinked with other lens proteins may also increase in *cryaa*-R49C mutant lenses [12].

**Fig. 3:**
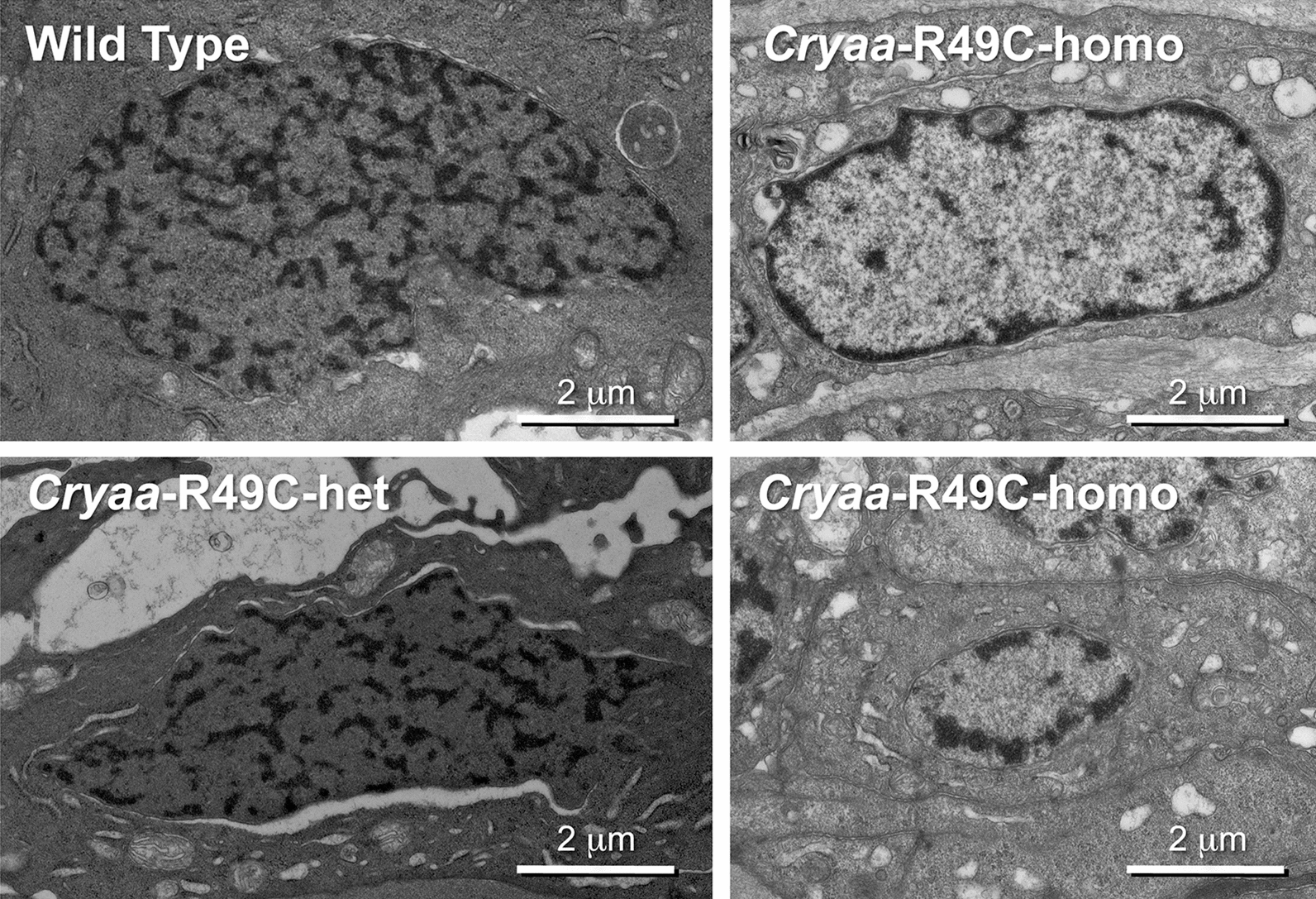
Electron micrographs of mouse lens epithelial nuclei. Wild type, *cryaa*-R49C het, and *cryaa*-R49C homo lenses were analyzed. Cells in the *cryaa*-R49C–het lenses appeared stressed, with swollen mitochondria, vacuoles and lacunae in these lenses. Cells in the *cryaa*-R49C-homo lenses had heterochromatin localized to the nuclear periphery

We used transmission electron microcopy to examine chromatin organization in the nuclei of lens epithelial cells and in newly differentiated fiber cells of WT and mutant mice (Fig. 3). Compared with WT or *cryaa*-R49C-het mice, the nuclei in *cryaa*-R49C-homo lens epithelial cells had markedly increased heterochromatin in a thin ring at the nuclear periphery and one or two condensations in the inner nucleus. We quantified the percentage of total number of nuclei with heterochromatin largely confined to the periphery in WT, *cryaa*-R49C-het, and *cryaa*-R49C-homo lens epithelial cells. We found that 72% of the nuclei in the homo group had heterochromatin (dark areas) largely confined to the periphery; the inner nuclei consisted mostly of euchromatin. In the *cryaa*-R49C–het lenses, 3% of the nuclei had this type of abnormal heterochromatin distribution. There were no nuclei with this type of heterochromatin distribution in the WT group. Like the WT lenses, *cryab*-R120G mutant lenses (both het and homo) did not show this type of chromatin reorganization (Additional file 7: Figure S5).

## Discussion

Here, we partly characterized the nature of histones and of chromatin changes that affected the observed alterations in histone expression in *cryaa*-R49C and *cryab*-R120G mutant mouse lenses. We identified changes in the relative intensity ratios of histones H2A/H2B and H3/H4. The results suggested that histone levels were abnormal in the lenses with cataract. The decreases in histone H2A/H2B and H3/H4 ratios suggested that the histone dimers did not form correctly in the mutant lens nuclei. These results are consistent with a study finding that mutation in histone cluster two h3 gene results in abnormal lens vesicle formation in mutant mice with apoptosis of the cells and eventual aphakia with retinal and corneal abnormalities. Similar findings were reported during zebrafish development in response to morpholino treatment and were associated with cataracts in mice [17].

Analysis of the histones extracted from adult mouse lenses from the knock-in mutants indicated that histones may undergo modifications in these mutant lenses. We have not identified the modifications present in the various peaks in the present study. Our previous proteomic results on proteins separated from neonatal WT and *cryaa*-R49C mutant lenses indicate that histones and α-crystallins are in the same locations [12]. Thermodynamically favored interactions between histones and α-crystallin have also been reported [20]. A possible function for histone-crystallin interactions inside the lens nuclei is due to the localization of *cryaa*-R49C mutant protein in the nucleus of transfected lens epithelial cells expressing this mutant [7].

The nuclear redistribution of heterochromatin suggested a drastic change in histones occurred in the *cryaa*-R49C-homo lens. The condensed, transcription-poor heterochromatin was organized throughout the nucleus in the WT and *cryaa*-R49C-het lenses. In contrast, the less condensed (lighter) euchromatin containing transcription factors, activated RNA polymerase, splicing machinery, and nascent RNA transcripts were all localized in the center of the nucleus. This characteristic indicated this location was the site of active gene transcription in the *cryaa*-R49C-homo lenses. There were a few clusters of dark heterochromatin in some of the *cryaa*-R49C-homo lens nuclei. The heterochromatin was also anchored to the periphery of the homo nuclei; this type of anchoring has been linked to lamin A/C and lamin B receptors. There are also models of retinal photoreceptors in which the nuclear distribution of chromatin is controlled by transcription factors [19].

Various mutations in the *cryaa* gene are associated with human cataract [25]. Future work should investigate whether changes in histone composition of the lens occurs in any of the other mutant lenses in vivo. A decrease in histone H3 observed in *cryaa*-R49C homo lenses indicated an important role of this histone in the lens. Histone H3 alterations are associated with defects in lens model systems [16, 17, 26].

## Limitations

The histone extraction procedures used in this study may not maintain the posttranslational modifications of histones (e.g. acetylation and methylation). The amount of histones extracted from individual mouse lenses is very low, particularly from the *cryaa*-R49C-homo lenses, due to their small size.

## Supplementary information

**Additional file 1: Table S1.** Relative intensities of histones extracted from mouse lenses (related to Figs. 1, 2, and Figs. S1–S3 and Table S2). The variability in the *m/z* ratios for histone components of the different mouse models suggest the presence of significant modifications in the various histone peaks [21].

**Additional file 2: Table S2.** Quantitative analysis of histones extracted from mouse lenses (related to Figs. 1, 2, and Figs. S1–3 and Table S1).

**Additional file 3: Figure S1.** MALDI-TOF MS analysis of histones isolated from *cryaa*-R49C-het mouse lenses (related to Fig. 1).

**Additional file 4: Figure S2.** MALDI-TOF MS analysis of histones isolated from *cryab*-R120G-het mouse lenses (related to Table S1).

**Additional file 5: Figure S3.** MALDI-TOF MS analysis of histones isolated from *cryab*-R120G-homo mouse lenses (related to Table S1).

**Additional file 6: Figure S4.** SDS-PAGE and immunoblotting of histones extracted from mouse lenses. (A) Coomassie blue-stained gel of histones from bovine lenses used as a control standard (lanes 1 and 2); WT mouse lenses (lane 3). Molecular weight markers (lane 4). (B) Immunoblot for the gel shown in (A) using a histone H2B antibody. Control standard (lanes 5 and 6); WT mouse lens (lane 7). (C) Coomassie blue-stained gel of histones from *cryaa*-R49C-het lenses (lane 8). (D) Immunoblot for the gel shown in (C) using a histone H2B antibody. (E) Coomassie blue-stained molecular weight markers on the membrane in (D). Note the increase in a band at ~ 17 kDa in the Coomassie stained gel and immunoblot in *cryaa*-R49C-het lenses as compared with WT. This band appears at a doublet, and both bands are present in the WT mice, although Coomassie-stained band at ~ 17 kDa did not appear in the immunoblot of WT lens histone preparation. The increase in the band intensity of the immunoblot at ~ 17 kDa in the *cryaa*-R49C-het mutant lenses suggested that the amount of highly modified histones increase the mutant lenses. The immunoblot analysis was performed using antibodies to histone H2B. The protein bands were visualized and quantified using an Odyssey system [20, 27]. Mouse monoclonal antibody to histone H2B (05-1352 clone 5HH2-2A8, lot 2495644; Millipore Corporation) was diluted to 1:700. Secondary donkey anti-mouse antibody was used at a 1:1000 dilution.

**Additional file 7: Figure** **S5.** Electron micrographs of mouse lens epithelial nuclei. Wild type, *cryab*-R120G het, and *cryab*-R120G homo lenses were analyzed. The *cryab*-R120G mutant lenses appear to have early stages of chromatin condensation compared with WT (Fig. 3).

## Data Availability

All data generated or analyzed during this study are included in this published article [and its additional files].

## Abbreviations

EM: Electron microscopy
Het: Heterozygous mutant
Homo: Homozygous mutant
MALDI-TOF: Matrix-assisted laser desorption/ionization-time of flight
SDS-PAGE: Sodium dodecyl sulfate polyacrylamide gel electrophoresis
WT: Wild-type
