# Supplementary material for: Changes in relative histone abundance and heterochromatin in αA-crystallin and αB-crystallin knock-in mutant mouse lenses
Source: BMC Res Notes. 2020 Jul 2;13:315. doi: 10.1186/s13104-020-05154-7 (PMC7331185; doi:10.1186/s13104-020-05154-7)
Supplement: Supplementary file 1 — Additional file 1: Table S1. Relative intensities of histones extracted from mouse lenses (related to Figs. 1, 2, and Figs. S1–S3 and Table S2). The variability in the m/z ratios for histone components of the different mouse models suggest the presence of significant modifications in the various histone peaks [21]. [file 13104_2020_5154_MOESM1_ESM.docx]

**Supplementary Table 1.** Relative intensities of histones extracted from mouse lenses (related to Figures 1, 2, Table 1 and Supplementary Figure 1-3)
