# Supplementary material for: Changes in relative histone abundance and heterochromatin in αA-crystallin and αB-crystallin knock-in mutant mouse lenses
Source: BMC Res Notes. 2020 Jul 2;13:315. doi: 10.1186/s13104-020-05154-7 (PMC7331185; doi:10.1186/s13104-020-05154-7)
Supplement: Supplementary file 2 — Additional file 2: Table S2. Quantitative analysis of histones extracted from mouse lenses (related to Figs. 1, 2, and Figs. S1–3 and Table S1). [file 13104_2020_5154_MOESM2_ESM.docx]

**Supplementary Table 1.** Relative intensities of histones extracted from mouse lenses* (related to Figures 1, 2, and Supplementary Figures 1-3).

*Histone H2 peak intensity represents the sum of peak intensities of [H2/2]^2+^ and H2. *Histones H3 peak intensity represents the sum of peak intensities of [H3/2]^2+^ and H3. **The histone percentage was calculated as the peak height of the histone(s)/sum of total peak intensity in the chromatogram × 100. Note that the H2A/H2B and H3/H4 ratios of relative intensity were nearly 1.0 in WT lens histones. Decrease in H3/H4 ratio in some genotypes was due to a decrease in the relative proportion of H3. n.d.*** indicates H2A could not be detected in histones extracted from the *cryab*-R120G mutant lenses.
