# Supplementary figures and images for: Changes in relative histone abundance and heterochromatin in αA-crystallin and αB-crystallin knock-in mutant mouse lenses
Source: BMC Res Notes. 2020 Jul 2;13:315. doi: 10.1186/s13104-020-05154-7 (PMC7331185; doi:10.1186/s13104-020-05154-7)

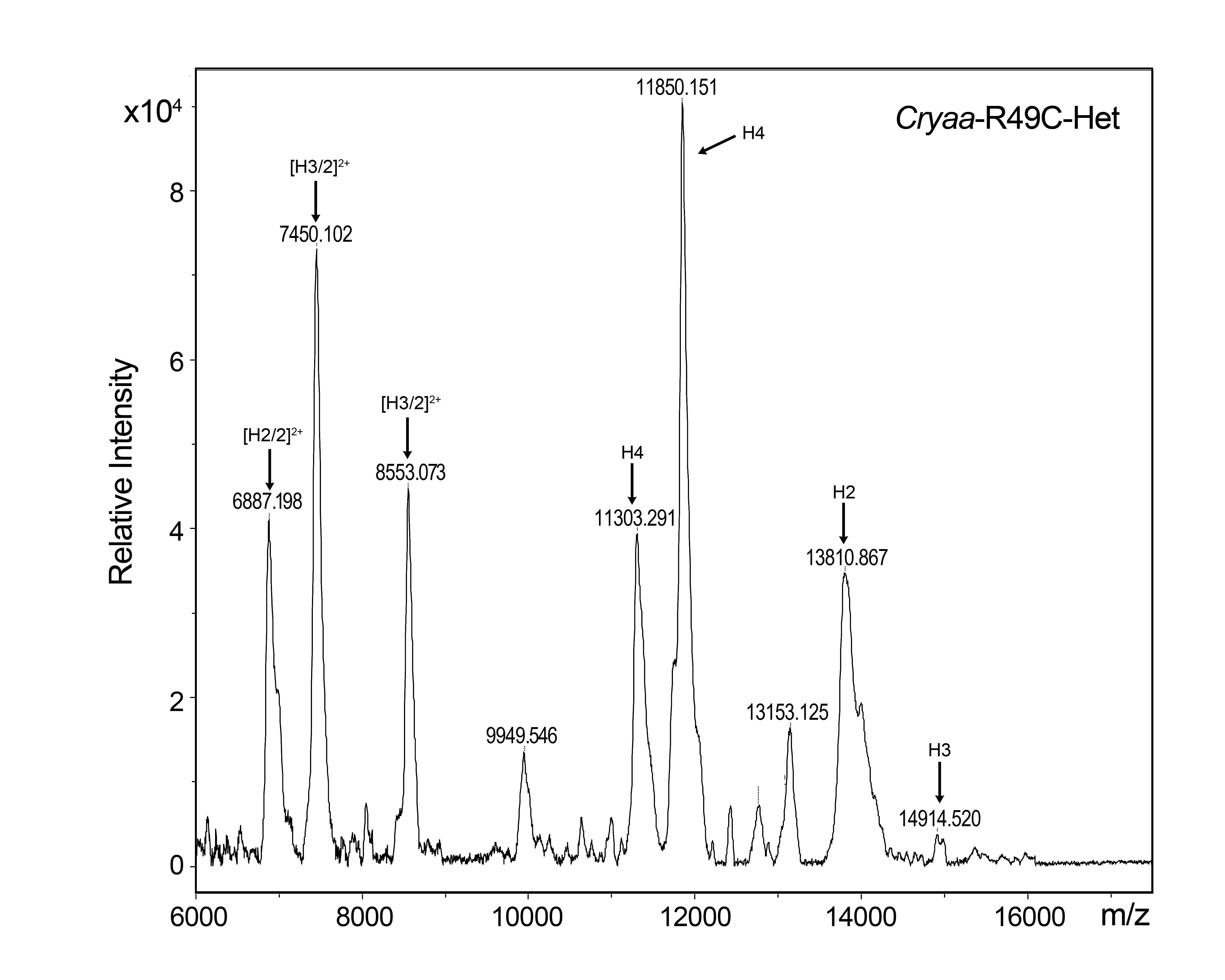

Supplement: Supplementary file 3 — Additional file 3: Figure S1. MALDI-TOF MS analysis of histones isolated from cryaa-R49C-het mouse lenses (related to Fig. 1). [file 13104_2020_5154_MOESM3_ESM.tif]

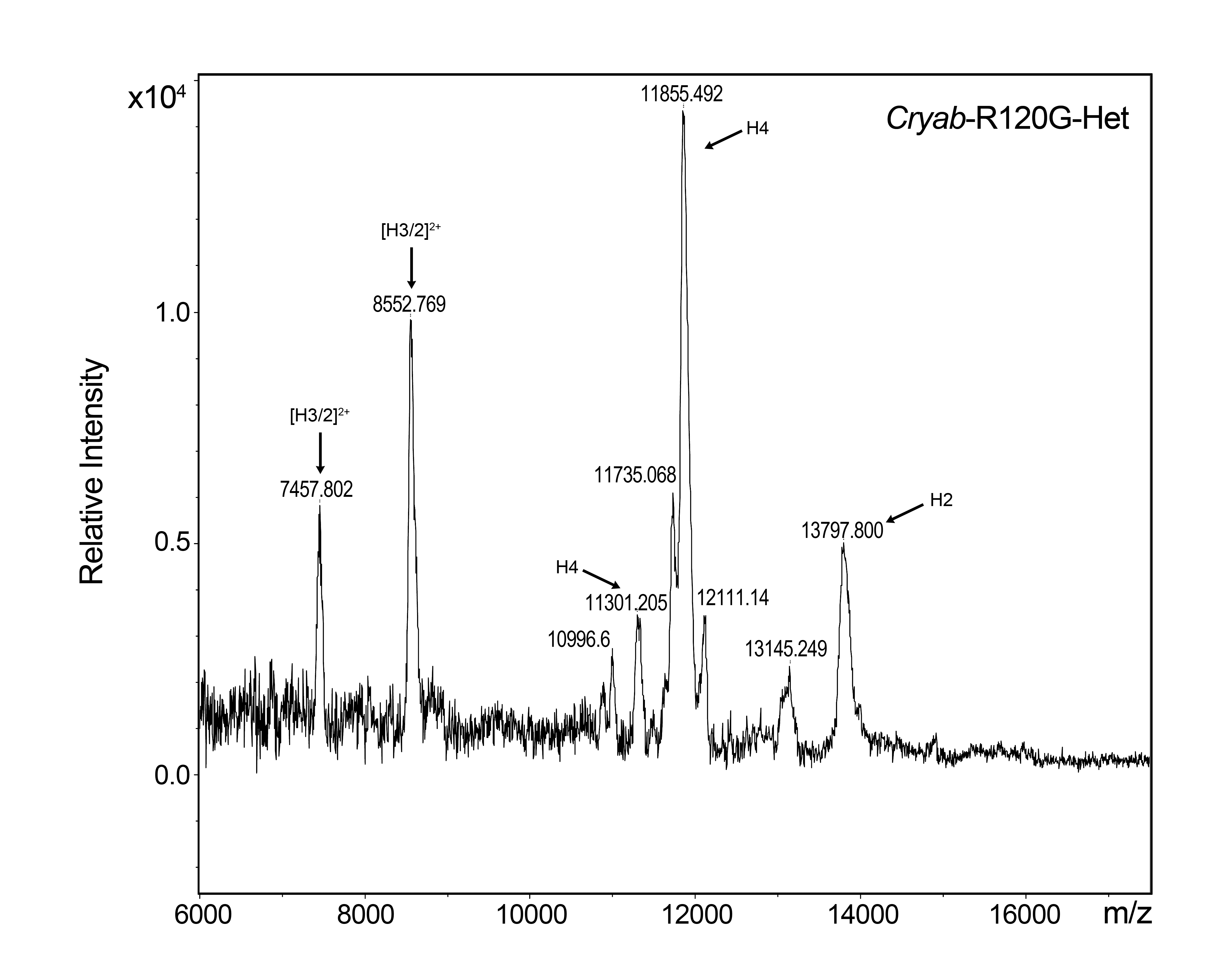

Supplement: Supplementary file 4 — Additional file 4: Figure S2. MALDI-TOF MS analysis of histones isolated from cryab-R120G-het mouse lenses (related to Table S1). [file 13104_2020_5154_MOESM4_ESM.tif]

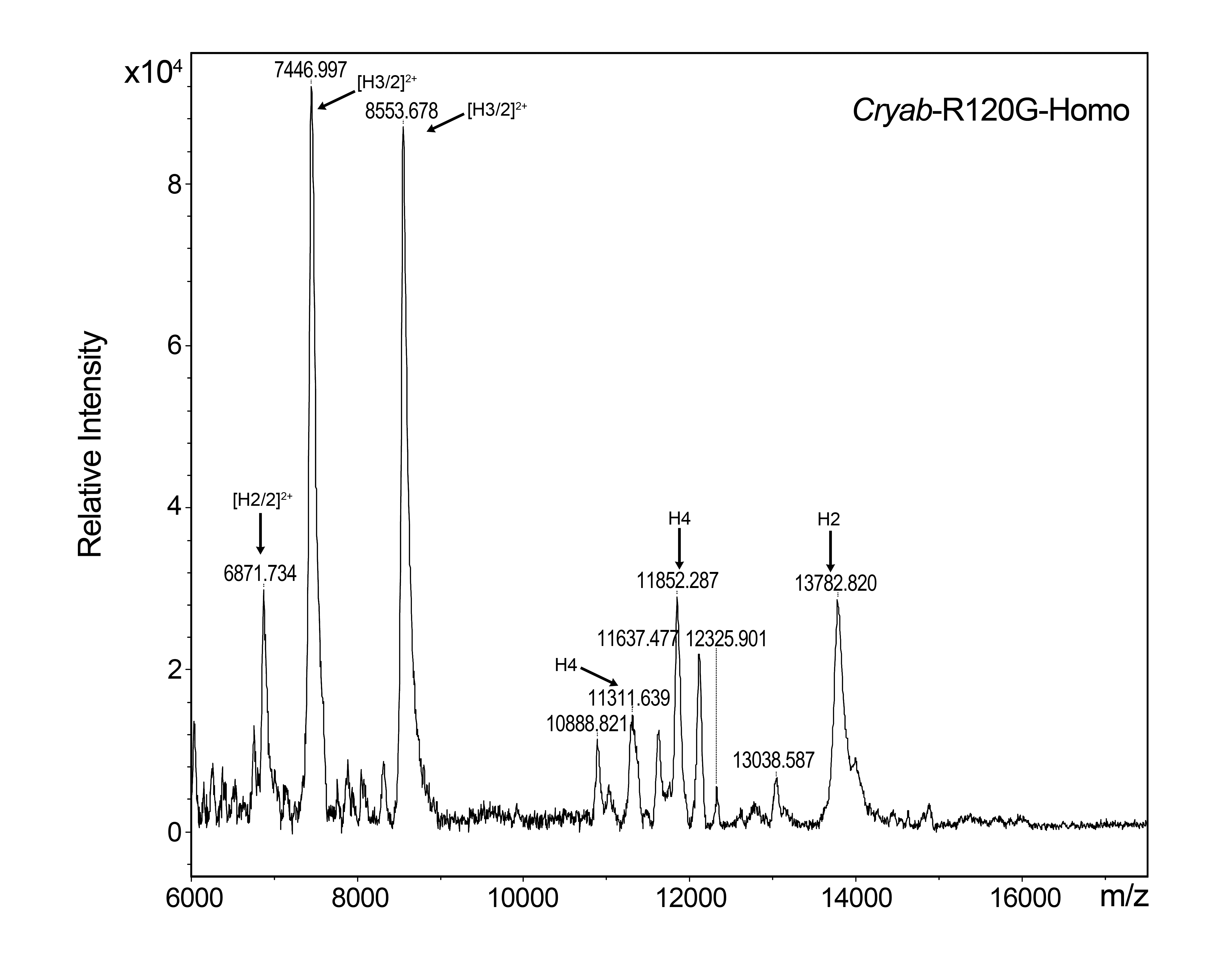

Supplement: Supplementary file 5 — Additional file 5: Figure S3. MALDI-TOF MS analysis of histones isolated from cryab-R120G-homo mouse lenses (related to Table S1). [file 13104_2020_5154_MOESM5_ESM.tif]

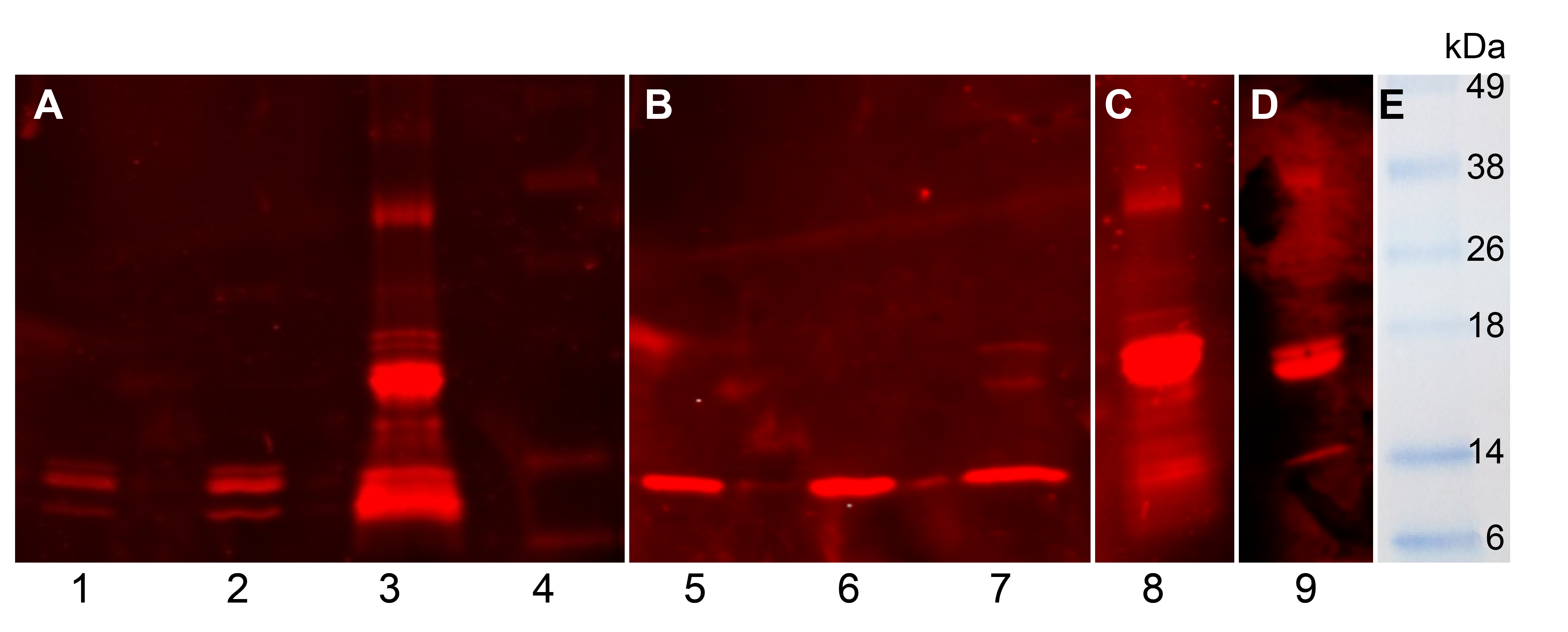

Supplement: Supplementary file 6 — Additional file 6: Figure S4. SDS-PAGE and immunoblotting of histones extracted from mouse lenses. (A) Coomassie blue-stained gel of histones from bovine lenses used as a control standard (lanes 1 and 2); WT mouse lenses (lane 3). Molecular weight markers (lane 4). (B) Immunoblot for the gel shown in (A) using a histone H2B antibody. Control standard (lanes 5 and 6); WT mouse lens (lane 7). (C) Coomassie blue-stained gel of histones from cryaa-R49C-het lenses (lane 8). (D) Immunoblot for the gel shown in (C) using a histone H2B antibody. (E) Coomassie blue-stained molecular weight markers on the membrane in (D). Note the increase in a band at ~ 17 kDa in the Coomassie stained gel and immunoblot in cryaa-R49C-het lenses as compared with WT. This band appears at a doublet, and both bands are present in the WT mice, although Coomassie-stained band at ~ 17 kDa did not appear in the immunoblot of WT lens histone preparation. The increase in the band intensity of the immunoblot at ~ 17 kDa in the cryaa-R49C-het mutant lenses suggested that the amount of highly modified histones increase the mutant lenses. The immunoblot analysis was performed using antibodies to histone H2B. The protein bands were visualized and quantified using an Odyssey system [20, 27]. Mouse monoclonal antibody to histone H2B (05-1352 clone 5HH2-2A8, lot 2495644; Millipore Corporation) was diluted to 1:700. Secondary donkey anti-mouse antibody was used at a 1:1000 dilution. [file 13104_2020_5154_MOESM6_ESM.tif]

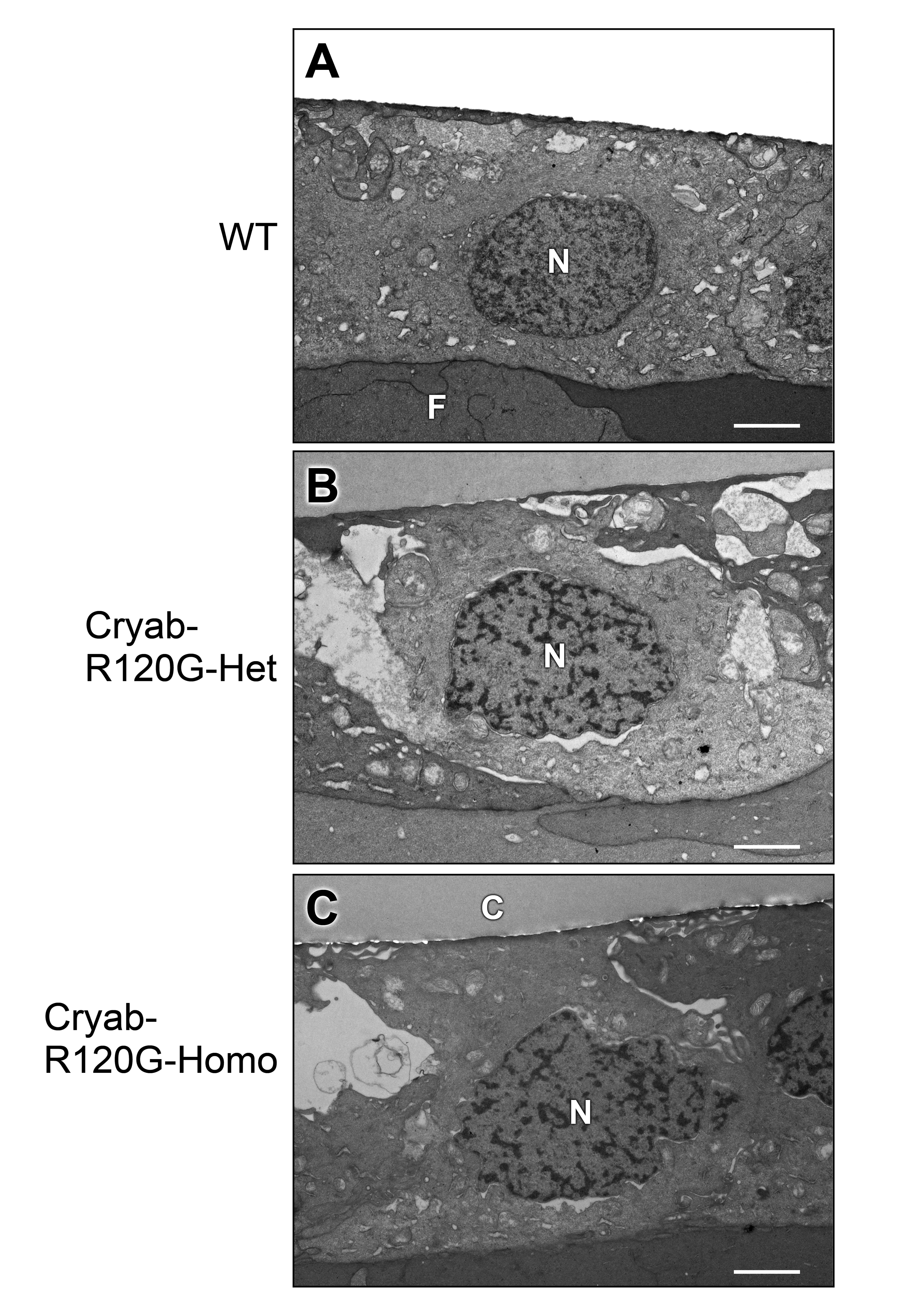

Supplement: Supplementary file 7 — Additional file 7: Figure S5. Electron micrographs of mouse lens epithelial nuclei. Wild type, cryab-R120G het, and cryab-R120G homo lenses were analyzed. The cryab-R120G mutant lenses appear to have early stages of chromatin condensation compared with WT (Fig. 3). [file 13104_2020_5154_MOESM7_ESM.tif]
